# Effects of Fiber Orientation on the Bearing Strength of 3D-Printed Composite Materials Produced by Fused Filament Fabrication

**DOI:** 10.3390/polym16243591

**Published:** 2024-12-22

**Authors:** Jun-Seok Oh, Min-Jae Oh, Zhiqiang Han, Hyoung-Seock Seo

**Affiliations:** 1 School of Naval Architecture & Ocean Engineering, University of Ulsan, Ulsan 44610, Republic of Korea; bh21007@ulsan.ac.kr (J.-S.O.); minjaeoh@ulsan.ac.kr (M.-J.O.); 2 School of Naval Architecture and Maritime, Zhejiang Ocean University, Zhoushan 316022, China; 3 Department of Autonomous Vehicle System Engineering, Chungnam National University, Daejeon 34134, Republic of Korea

**Keywords:** three-dimensional printing, fused filament fabrication, fiber orientation, bearing strength, failure mode

## Abstract

Among 3D printing technologies, fused filament fabrication (FFF) is a fast, simple, and low-cost technology that is being explored in a variety of industries. FFF produces composites using thermoplastic filaments, limiting the applicability of welding. Therefore, mechanical fastening is required to join FFF composites with metals or dissimilar materials. The strength characteristics of fastened joints vary with fiber orientation, necessitating further research. Additionally, in the case of FFF, the strength trends may differ from those of traditional composites due to the voids and curved surfaces formed during the process. In this study, 3D-printed composite specimens with seven different fiber orientations were fabricated using the Markforged X7™ printer. The bearing strength and failure modes were analyzed as a function of fiber orientation. Unlike traditional composites, specimens with a ±15° fiber orientation exhibited a 7.56% higher bearing strength compared to those with a 0° orientation. However, the fracture energy of the ±15° specimens was 39.56% lower. Specimens with fiber orientations between 0° and ±45° primarily showed bearing failure modes, while those with orientations from ±60° to 90° exhibited net-tension failure modes. These results confirm that when using manufacturing methods like FFF, the strength trends vary with fiber orientation compared to traditional composites. Further research is necessary to optimize fiber orientation and improve structural performance.

## 1. Introduction

Three-dimensional printing is an additive manufacturing (AM) technology that uses three-dimensional (3D) model data to fabricate a variety of structures and complex geometries [1]. Among AM technologies, fused filament fabrication (FFF), also known as fused deposition modeling (FDM), is one of the most widely studied and utilized 3D printing methods in various industries due to its advantages of speed, simplicity, and low cost [2]. However, AM technology still faces several challenges during the transition from CAD to 3D-printed parts, including defects caused by tessellation, especially on curved surfaces [3], and poor mechanical performance due to voids [4,5].

FFF involves depositing multiple layers of molten thermoplastic filaments through a heated nozzle and can process a wide range of materials, including polylactic acid (PLA), metal–polymer composites, fiber-reinforced polymer composites, ceramic materials, and more [6]. Among these, fiber-reinforced polymer composites typically consist of a matrix, such as nylon, and reinforcements, such as carbon fibers, glass fibers, and Kevlar fibers. Among the reinforcements, carbon fiber is particularly notable for its high strength and stiffness compared to other fibers, as well as its resistance to chemical degradation, making it an essential form of reinforcement for fiber-reinforced plastic composites [7].

To apply composites made by FFF in structures, it is necessary to connect them with metallic or dissimilar materials. Common methods for structural connections include welding, adhesive bonding, and mechanical fastening [8]. For composites made by FFF, welding is considered inappropriate because the materials used are generally thermoplastic. Therefore, adhesive bonding or mechanical fastening is typically used. Adhesive bonding can reduce the weight of the structure and simplify the design [9], but it requires surface pretreatment and is highly susceptible to environmental factors such as humidity and temperature [10]. However, mechanical fasteners are often used to join composites because they do not require surface pretreatment, are not sensitive to humidity or temperature, and offer advantages in maintenance [11].

Mechanical fastening strength is influenced by parameters such as geometric properties and fiber orientation [12,13]. In particular, unlike metallic materials, composites exhibit anisotropic properties, making fiber orientation a critical factor to consider [14]. Furthermore, since bearing strength and failure modes vary depending on fiber orientation, research on this topic is essential. Previous studies on fiber orientation include that conducted by İçten and Sayman [15], who investigated the relationship between fiber orientation and bearing strength when the composites had fiber orientations of 0°, 30°, and 45°, and observed that the composites exhibited maximum bearing strength at 0° and minimum bearing strength at 45°. Additionally, within the same geometric dimensions, the failure mode remained consistent despite changes in fiber orientation. Zhang et al. [16] predicted the failure mode as a function of fiber orientation in 15° increments from 0° to 90° using the equation proposed by Chang, which is used for the failure prediction of composite mechanical joints. Giorleo et al. [17] studied the mechanical behavior of pin bearings in specimens fabricated by Markforged’s continuous filament fabrication (CFF). The results showed that 0° and 45° exhibited comparable bearing strength, with 90° having the lowest value.

In summary, research on fiber orientation has primarily been performed in 30° or 45° increments, and research on finer granularity is scarce. In particular, composites made with the FFF process require further research because challenges such as voids and curved surfaces during the printing process may result in different bearing strengths and failure modes depending on fiber orientation.

Therefore, in this study, we investigated the bearing strength of 3D-printed composite specimens fabricated using the FFF process based on fiber orientation. To achieve a finer analysis compared to previous studies, we divided the fiber orientation into 15° increments, resulting in seven fiber orientations from 0° to 90°. Through this approach, we confirmed that bearing strength trends differ between traditional composites and 3D-printed composites, and we analyzed the causes using Eiger software and Micro CT. Furthermore, we examined the failure mode trends through microscopic analysis.

## 2. Materials and Methods

### 2.1. Materials

The specimen materials used in this study were fabricated using Markforged’s continuous carbon fiber filament and the Onyx filament to achieve high-strength specimens (Markforged, Waltham, MA, USA). Continuous carbon fiber is known for its high strength-to-weight ratio, making it a widely used reinforcement in composite materials [6,7]. Markforged’s continuous carbon fiber offers superior strength compared to conventional carbon fibers, enhancing structural performance. Onyx is a nylon-based filament reinforced with micro-carbon fibers, providing higher strength and stiffness than pure nylon. The material properties of carbon fiber and Onyx are presented in Table 1.

### 2.2. Manufacturing Process

The specimens in this paper were fabricated using Markforged’s X7™ printer (Markforged, Waltham, MA, USA). The Markforged X7™ is based on continuous filament fabrication (CFF) technology, which is a specialized method that expands on the FFF technology developed by Markforged.

**Figure 1:**
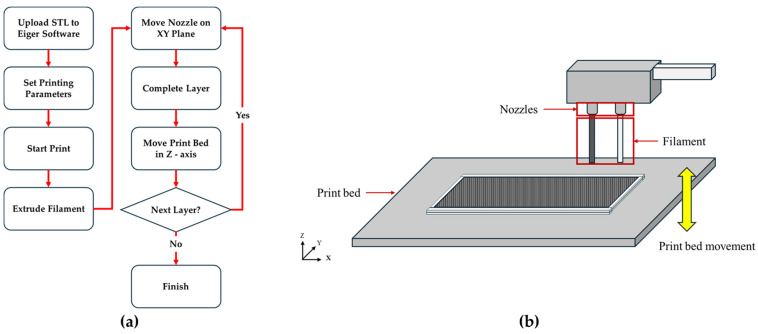
Overview of the Markforged printing process: (**a**) flow chart of the printing process; (**b**) schematic of the printing method.

The Markforged printer uploads the STL file to the Eiger software, sets the materials, fill pattern, wall layer, fiber pattern type, concentric fiber rings, and fiber angles, and then proceeds to print. During the printing process, two heated nozzles move along a predefined path on the XY plane, extruding the filament in a semi-liquid state to laminate each layer. Once a layer is completed, the print bed moves in the Z-axis direction to set the height for the next layer, and the print head resumes laminating along the new XY plane. This process is repeated layer by layer to build the entire structure, with the filament being continuously deposited without interruption (Figure 1).

### 2.3. Specimen

**Figure 2:**
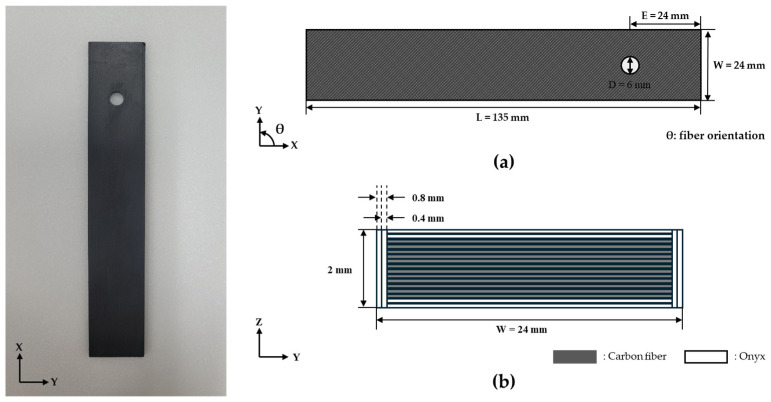
Specimen dimensions in XY and YZ planes: (**a**) XY plane; (**b**) YZ plane (cross-section).

Figure 2 shows the actual specimen along with the specimen dimensions in the xy and yz planes, with detailed dimensions summarized in Table 2. The specimen length (L), hole diameter (D), and thickness (t) were set to 135 mm, 6 mm, and 2 mm, respectively, following ASTM D5961 [19]. To achieve a specimen thickness of 2 mm, the specimen was composed of a total of 16 layers, as the Markforged printer has a fixed layer thickness of 0.125 mm. The specimen width (W) and edge distance (E) were both set to 24 mm to achieve the W/D and E/D ratios of four, optimizing the bearing strength [20,21]. For the outer wall layer, Onyx was used, consisting of two layers with a thickness of 0.4 mm per layer.

The top and bottom layers of the specimen were fabricated using Onyx with a fixed fiber orientation of [±45°] determined by the Markforged printing process. The core layers were reinforced with continuous carbon fibers, and as shown in Table 3, fiber orientations were selected at 15° increments from 0° to 90°, resulting in seven orientations. This selection was motivated by the fact that previous studies typically used 30° or 45° increments, indicating a need for research on finer increments. Furthermore, strength studies on specimens produced by the FFF method showed higher tensile strength at 15° rather than at 0°, which supported the choice of these specific fiber orientations [22].

### 2.4. Test Setup

**Figure 3:**
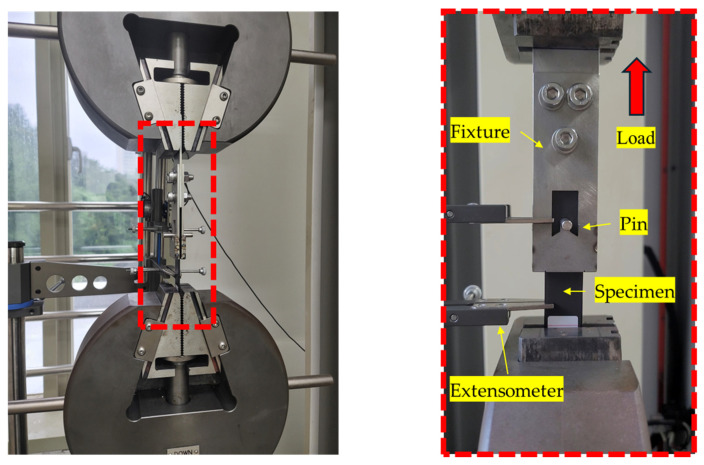
Explanation of bearing test setup.

The bearing strength of the specimens was evaluated using a DTU-900MH300kN universal testing machine (Daekyung Tech, Incheon, Republic of Korea) with a 300 kN load cell. A 6 mm pin and a double-lap fixture were fabricated from stainless steel to apply the bearing load to the hole. The specimen was setup and tested, as shown in Figure 3. The tests were performed according to ASTM D5961 at a speed of 2 mm/min, with each fiber orientation tested five times. Failure was defined as occurring when the load reached 70% of the maximum load.

### 2.5. Calculation of Bearing Strength, Bearing Strain and Fracture Energy

The ultimate bearing strength and bearing strain were calculated according to ASTM D5961. Equation (1) is the expression for ultimate bearing strength ($F^{bru}$):

(1) $$F^{bru}=\frac{P^{max}}{\left(k\times D\times h\right)}$$

where $F^{bru}$ is the ultimate bearing strength (MPa). *P* is the maximum force prior to failure (N). *k* is the force per hole factor, which is 1.0 for single-fastener or pin tests, 2.0 for double-fastener tests, and 1.0 was used in this experiment. *D* is the hole diameter (mm), and *h* is the specimen thickness (mm). Equation (2) is the expression for the bearing strain ($\varepsilon^{br}$):

(2) $$\varepsilon^{br}=\frac{\delta }{\left(K\times D\right)}$$

where $\varepsilon^{br}$ is the bearing strain (mm/mm). δ is the displacement (mm) measured by the extensometer. *K* is a constant, 1.0 for double-shear tests and 2.0 for single-shear tests, and we used 1.0 in this experiment.

**Figure 4:**
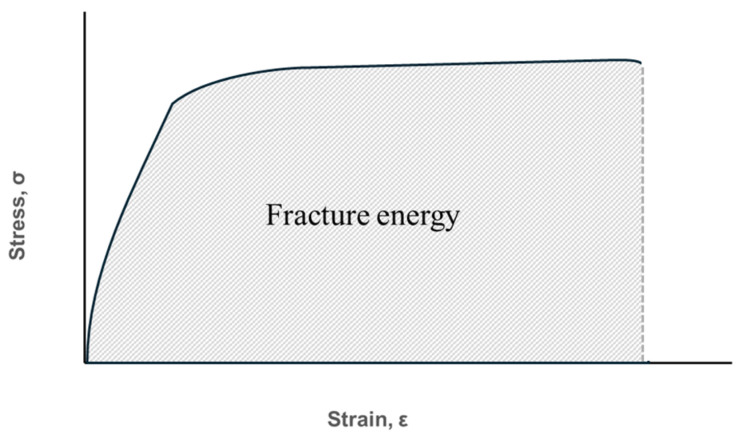
Explanation of fracture energy.

Fracture energy refers to the total energy that a specimen can absorb to resist fracture, and it can be calculated using the area under the stress–strain curve, as shown in Figure 4.

## 3. Experiment Results and Discussion

### 3.1. Mechanical Properties

**Figure 5:**
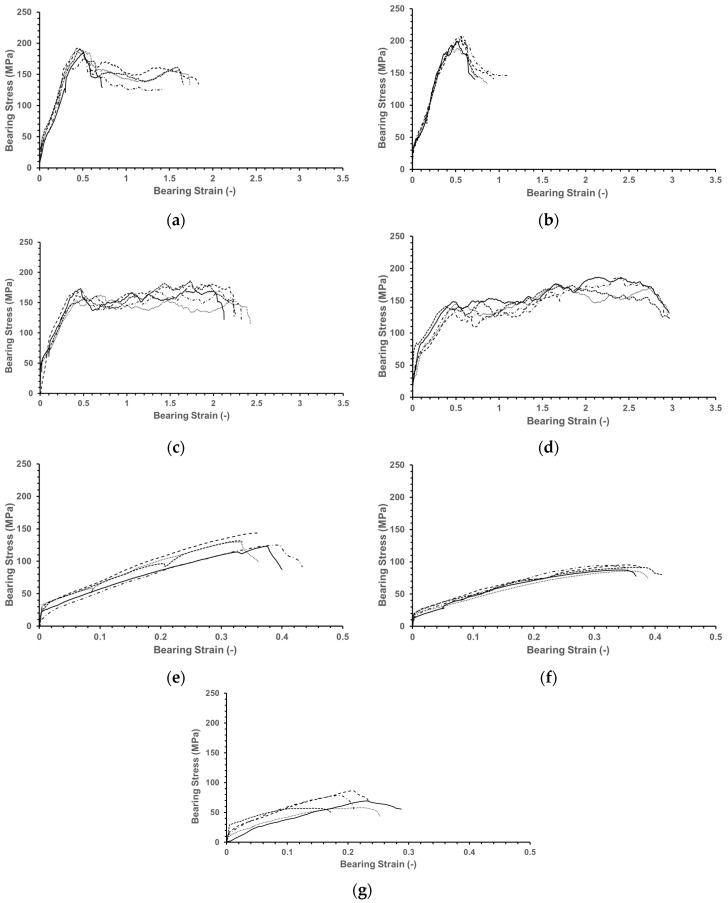
Bearing stress–strain curve for different specimen fiber orientations tested with five samples each: (**a**) Specimen (0°); (**b**) Specimen (±15°); (**c**) Specimen (±30°); (**d**) Specimen (±45°); (**e**) Specimen (±60°); (**f**) Specimen (±75°); and (**g**) Specimen (90°).

Figure 5 presents the bearing stress–strain curve, derived by converting the load–displacement data obtained from the tests into bearing stress and strain values using Equations (1) and (2). Table 4 summarizes the ultimate bearing strength, strain, and fracture energy as the mean ± standard deviation.

Generally, for traditional composites, the fiber orientation with the highest bearing strength is 0°, and the bearing strength tends to decrease as the fiber orientation becomes more perpendicular to the load direction. However, in this study, specimens fabricated using the CFF process exhibited a 14.1 MPa (7.56%) higher bearing strength for Specimen (±15°) compared to Specimen (0°). This result is likely due to the influence of the voids generated during the printing process and the U-shaped fiber turning regions.

**Figure 6:**
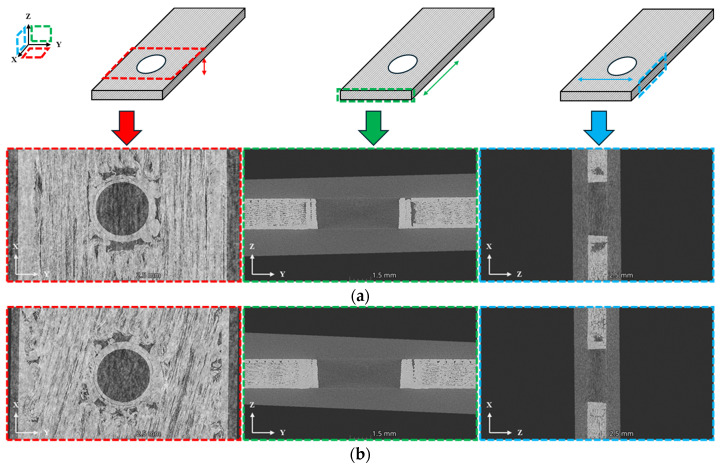
Micro CT image: (**a**) Specimen (0°); (**b**) Specimen (±15°).

Figure 6 shows the micro-CT images of 0° and ±15° fiber orientations, which reveal the voids generated during the printing process. As shown in Figure 6, the size and number of voids around the hole are larger and greater in the 0° fiber orientation compared to the ±15° fiber orientation. Voids present within the specimen cause stress concentrations at their locations, leading to reduced mechanical properties and the initiation of cracks [2,23]. Therefore, the 0° fiber orientation, which has larger and more numerous voids, is more susceptible to initial fracture than the ±15° fiber orientation.

**Figure 7:**
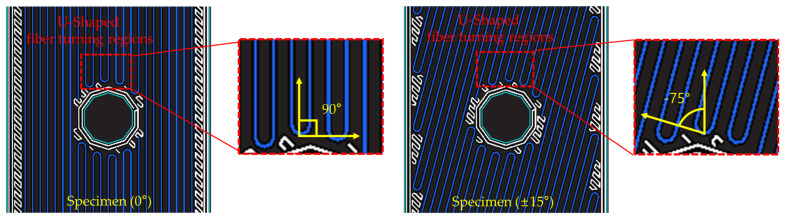
Fiber rotation section of 0° and 15° fiber orientations.

**Figure 8:**
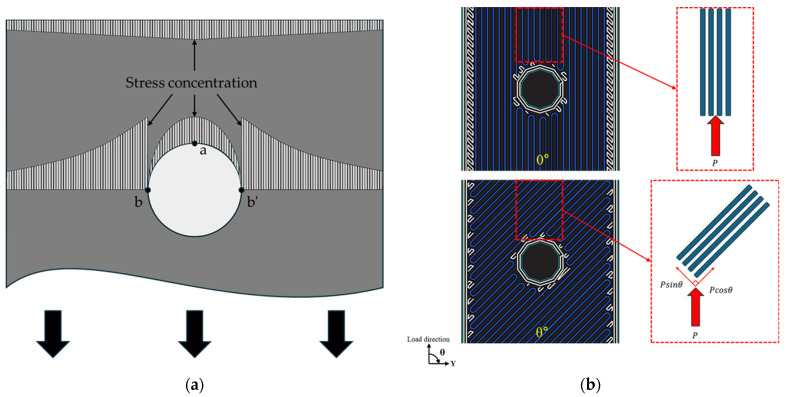
Explanation of stress concentration and stress decomposition: (**a**) stress concentration; (**b**) stress decomposition.

As shown in Figure 7, Specimen (0°) includes a 90° fiber turning region, while Specimen (±15°) includes a ±75° fiber turning region. The stress concentration around the hole under the bearing load is distributed, as shown in Figure 8a, and the fiber-turning regions experience high compressive stress due to stress concentration. At this point, the 90° fiber turning region is perpendicular to the load direction, failing to decompose the applied stress into longitudinal and transverse directions. In contrast, the ±75° fiber turning region allows the applied stress to be decomposed into longitudinal and transverse components. Explaining the stress decomposition based on fiber orientation in more detail using Figure 8b, the 0° fiber orientation directly absorbs the applied load P. However, for a θ° fiber orientation, the load P is decomposed into Psinθ and Pcosθ components, distributing the stress into longitudinal and transverse directions. This mitigates the stress concentration around the hole and reduces the occurrence and propagation of damage [24]. Therefore, due to the differences in fiber orientation in the U-shaped fiber turning regions, Specimen (0°) experiences initial fracture relatively faster than Specimen (±15°).

The onset of initial fracture in Specimen (0°) and Specimen (±15°) can be identified through the bearing stress–strain curve. Generally, the bearing stress–strain curve consists of a linear region and a nonlinear region, with the transition from linear to nonlinear caused by matrix cracking, fiber buckling, breakage, and delamination due to the applied loads [25,26]. Therefore, the strain value at which the curve transitions from the linear to the nonlinear region can indicate the onset of initial fracture. According to the bearing stress–strain curve, the nonlinear region begins at a strain of 0.47 for Specimen (0°), whereas it starts at a strain of 0.53 for Specimen (±15°). This indicates that fracture initiates earlier in the Specimen (0°).

**Figure 9:**
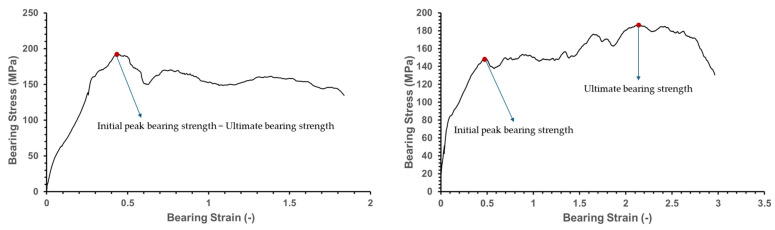
Explanation of bearing stress–strain curve.

Specimen (±30°) and Specimen (±45°) exhibit a lower initial peak bearing strength compared to Specimen (0°) and Specimen (±15°), which is attributed to the lower compressive and tensile strength of the ±30° and ±45° fiber orientations compared to the 0° and ±15° fiber orientations. However, Specimen (±30°) and Specimen (±45°), as shown in Figure 9, exhibit a higher ultimate bearing strength after the initial peak bearing strength compared to the other specimens. This is likely because, as the fiber orientation approaches ±45°, the stress is more evenly distributed into longitudinal and transverse directions, preventing critical specimen failure [12,27].

Specimen (±60°), Specimen (±75°), and Specimen (90°) exhibit a significant reduction in bearing strength and strain compared to the other specimens. This is attributed to the dominant influence of lower tensile strength over the ability to decompose the applied stress based on fiber orientation. Further details of this will be discussed in the failure mode analysis.

### 3.2. Failure Mode

**Figure 10:**
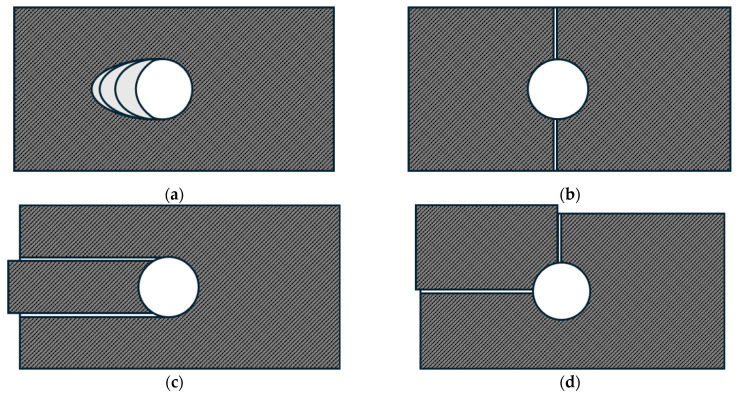
Failure modes: (**a**) bearing failure mode; (**b**) net-tension failure mode; (**c**) shear-out failure mode; and (**d**) cleavage failure mode.

Figure 10 shows the failure modes of a bearing test according to the ASTM D5961.

Bearing failure mode occurs when local pressure is applied to the contact area of a pin or bolt with a connection when the free edge distance and width of the specimen are sufficient [28]. The net-tension failure mode occurs when the width excluding holes is insufficient, or the tensile strength of the fiber is insufficient. The shear-out failure mode occurs when there is an insufficient free edge distance [24,25,28]. The cleavage failure mode is caused by the concentration of stress at the tip and free edge distance of the hole due to the bearing pressure exerted by the fastener and has the characteristics of net-tension and shear-out failure modes [28]. Net-tension, shear-out, and cleavage failure modes typically occur suddenly and are often catastrophic [24,29]. However, the bearing failure mode is generally desirable because it progresses slowly and provides ample warning before final failure [28,29].

**Figure 11:**
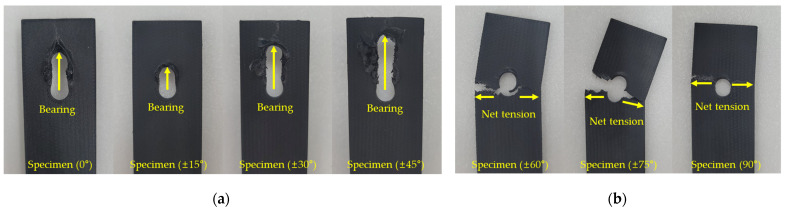
Typical failure modes of bearing test specimens: (**a**) specimen of bearing failure mode; (**b**) specimen of net-tension failure mode.

The typical failure modes of each specimen are shown in Figure 11, and their corresponding results are summarized in Table 5. All Specimens (0°), Specimens (±15°), and Specimens (±30°) exhibited bearing failure mode. For Specimen (±45°), four specimens exhibited bearing failure mode, while one exhibited both the bearing failure mode and net-tension failure mode. Specimens (±60°), Specimens (±75°), and Specimens (90°) all exhibited the net-tension failure mode.

**Figure 12:**
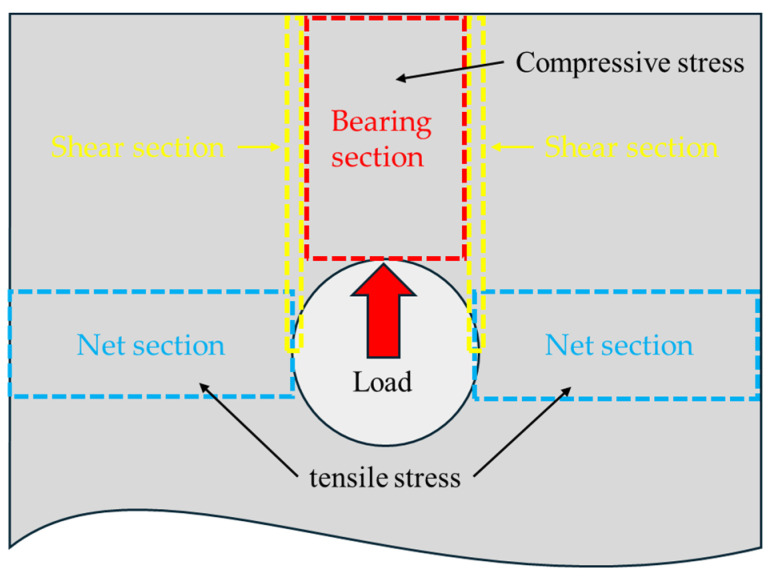
Stress section under bearing load.

Figure 12 shows the types of stress generated under a bearing load and the regions where these stresses act. During a bearing test, compressive stress is applied to the bearing section, as shown in Figure 12, while tensile stress acts on the net section. For Specimen (0°), Specimen (±15°), Specimen (±30°), and Specimen (±45°), which exhibit a bearing failure mode, the tensile strength of the fiber orientation is relatively high. As a result, failure predominantly occurs in the bearing section or shear section, where compressive stress acts, rather than in the net section, where tensile stress is applied. The specimens in this study were designed with a sufficiently high E/D ratio based on previous research, which prevented shear failure modes and resulted in bearing failure modes.

In contrast, Specimen (±60°), Specimen (±75°), and Specimen (90°), which exhibit pure tensile failure modes, have fiber orientations that are nearly perpendicular to the load direction. The tensile strength is highest when the fiber orientation is aligned with the load direction (0°) and decreases as the fiber orientation approaches 90°. Therefore, failure predominantly occurs in the net section, where tensile stress is applied.

#### 3.2.1. Bearing Failure Mode

**Figure 13:**
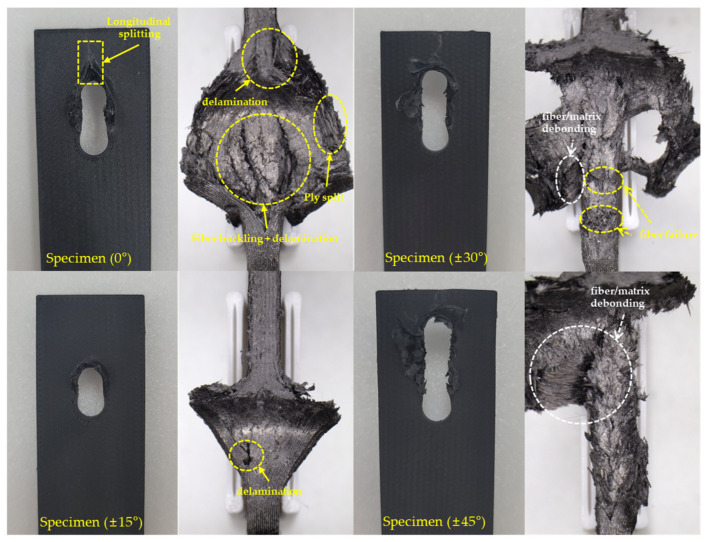
Fracture mode of bearing failure mode specimens.

**Figure 14:**
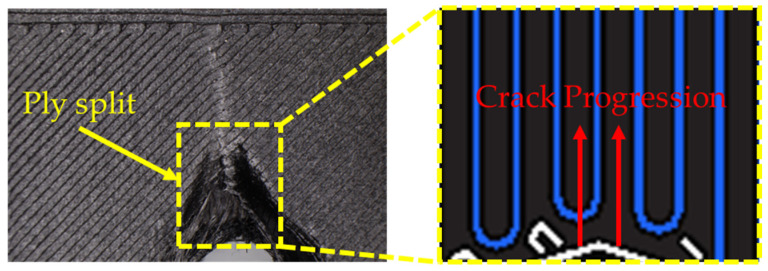
Ply split and crack progression of Specimen (0°).

Figure 13 shows a microscopic image of the cross-section of specimens exhibiting a bearing failure mode. Specimen (0°) displays ply splitting and delamination caused by fiber buckling in the longitudinal direction. Ply splitting is a phenomenon typically observed in specimens with a single-fiber orientation [30]. Specifically, for the CFF-fabricated 0° fiber orientation specimens, stresses act on the voids in the 0° direction caused by the U-shaped fiber turning regions, as shown in Figure 14. This initiates ply splitting within each layer, which propagates to the specimen’s edges and results in through-thickness ply splitting, as shown in Figure 14 [31]. For Specimen (±30°) and Specimen (±45°), fiber/matrix debonding was observed on the specimens’ surfaces, which is attributed to the influence of interlaminar shear stress [32].

#### 3.2.2. Net-Tension Failure Mode

**Figure 15:**
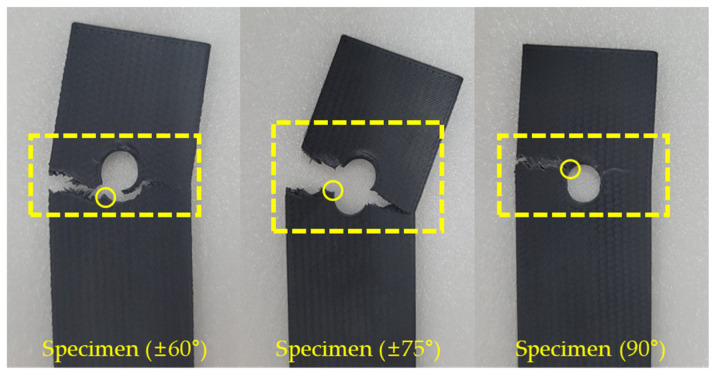
Specimen of net-tension failure mode.

**Figure 16:**
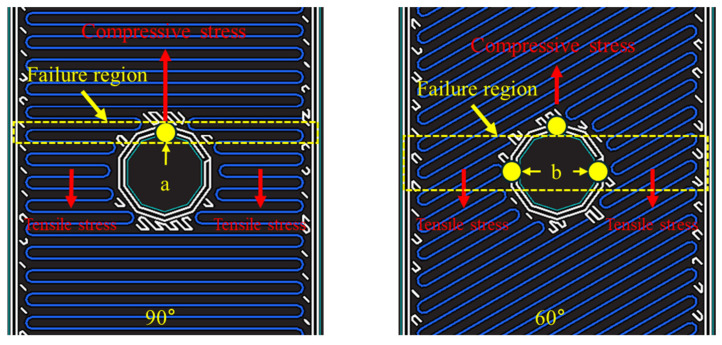
Failure region of 90° and 60° fiber orientation.

Specimen (±60°), Specimen (±75°), and Specimen (90°) exhibited a pure tensile failure mode due to their low tensile strength, as shown in Figure 15. As the fiber orientation became perpendicular to the load direction, failure occurred near the bearing section. In particular, Specimen (90°), which has the lowest tensile strength, could not resist the tensile stress in the net section induced by the bearing load and ultimately fractured along the specimen’s width at point a in the bearing section, where the highest stress occurred, as shown in Figure 16. Specimen (±60°) and Specimen (±75°) have relatively higher tensile strength than Specimen (90°). Therefore, failure did not occur at point a in Figure 16 due to the influence of tensile strength in the net section. Instead, as the tensile strength increased with the fiber orientation, the failure region shifted to point b.

### 3.3. Fracture Energy

**Figure 17:**
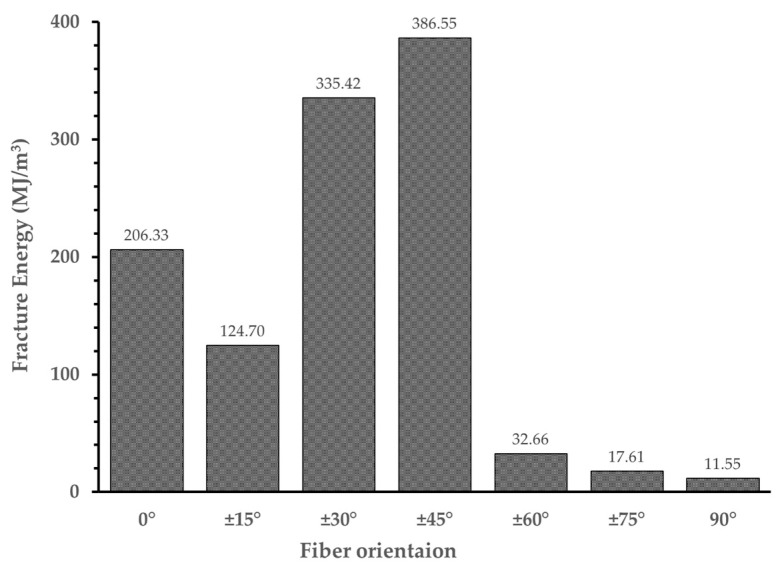
Fracture energy of specimens.

**Figure 18:**
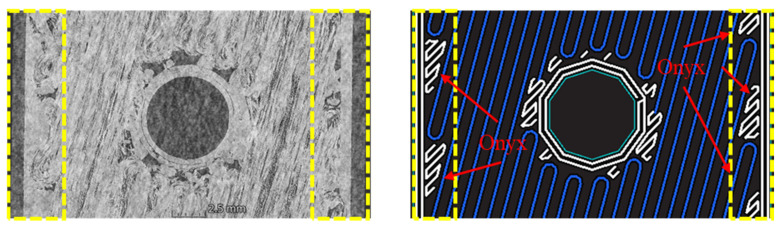
Voids and Onyx distribution at specimen edges for ±15° fiber orientation.

Figure 17 shows a bar chart illustrating the fracture energy of the specimens summarized in Table 4. Among the specimens exhibiting a bearing failure mode, including Specimen (0°), Specimen (±15°), Specimen (±30°), and Specimen (±45°), all specimens except for Specimen (±15°) showed an increase in fracture energy as the fiber orientation angle increased. This trend is attributed to the fact that as the fiber orientation increases from 0° to ±45°, the stress is uniformly decomposed, delaying the progression of failure and resulting in higher strain. However, for Specimen (±15°), the reduction in fracture energy is attributed to the presence of numerous voids at the specimen’s edges and a reduced fiber volume fraction caused by matrix regions formed during the printing process due to fiber orientation, as shown in Figure 18. For the specimens exhibiting pure tensile failure modes, including Specimen (±60°), Specimen (±75°), and Specimen (90°), the fracture energy decreased by 84.17% to 97.01% compared to those exhibiting a bearing failure mode. Additionally, the energy fracture decreased as the fiber orientation increased. This was due to the early initiation of ply splitting in the net section caused by the low tensile strength as the fiber orientation became more perpendicular to the load direction.

## 4. Conclusions

This study investigated the bearing strength of composite specimens in 3D-printed using the fused filament fabrication (FFF) method, and the results are as follows.

- Unlike traditional composites, specimens with ±15° fiber orientation exhibited a 7.56% higher bearing strength compared to those with 0° fiber orientations. This is attributed to the voids and U-shaped fiber-turning regions formed during the printing process.
- Specimens with ±45° fiber orientation showed the most uniform distribution of compressive and tensile stresses under the bearing loads, delaying severe initial damage and slowing the progression of failure.
- As the fiber orientation exceeded ±60° and approached 90°, which is perpendicular to the load direction, the low tensile strength of the fibers led to net tension failure.
- Specimens with ±15° fiber orientation exhibited 39.56% lower fracture energy compared to those with 0° fiber orientations due to the voids and lower fiber volume fraction at the specimen edges caused by the CFF process.

The results confirm that composite materials fabricated using the FFF method exhibit different bearing strength trends compared to traditional composites. Therefore, when employing new manufacturing processes like the FFF method, further research on optimizing fiber orientation can potentially enhance structural strength.

## Figures and Tables

**Table 1:** Carbon fiber and Onyx material properties by Markforged [18].

Properties	Carbon Fiber	Onyx
Test (ASTM)	D3039	D638
Tensile Modulus (GPa)	60	2.4
Tensile Strength (MPa)	800	40
Tensile Strain at Break (%)	1.5	25
Test (ASTM)	D790	D790
Flexural Strength (MPa)	540	71
Flexural Modulus (GPa)	51	3.0
Flexural Strain at Break (%)	1.2	-
Test (ASTM)	D6641	-
Compressive Strength (MPa)	420	-
Compressive Modulus (GPa)	62	-
Compressive Strain at Break (%)	0.7	-

**Table 2:** Dimensions of the specimens.

L (mm)	D (mm)	W (mm)	E (mm)	t (mm)	W/D	E/D
135	6	24	24	2	4	4

**Table 3:** Fiber orientation and the material of the specimens by layer.

Specimen Type	Material	Total Layer No.	Fiber Orientation
Specimen (0°)	Onyx	2	[±45°]
	Carbon fiber	12	[0°]_12_
	Onyx	2	[±45°]
Specimen (±15°)	Onyx	2	[±45°]
	Carbon fiber	12	[±15°]_6_
	Onyx	2	[±45°]
Specimen (±30°)	Onyx	2	[±45°]
	Carbon fiber	12	[±30°]_6_
	Onyx	2	[±45°]
Specimen (±45°)	Onyx	2	[±45°]
	Carbon fiber	12	[±45°]_6_
	Onyx	2	[±45°]
Specimen (±60°)	Onyx	2	[±45°]
	Carbon fiber	12	[±60°]_6_
	Onyx	2	[±45°]
Specimen (±75°)	Onyx	2	[±45°]
	Carbon fiber	12	[±75°]_6_
	Onyx	2	[±45°]
Specimen (90°)	Onyx	2	[±45°]
	Carbon fiber	12	[90°]_12_
	Onyx	2	[±45°]

**Table 4:** Result of ultimate bearing strength, bearing strain, and fracture energy.

Specimen Type	Ultimate Bearing Strength (MPa)	Bearing Strain (-)	Fracture Energy (MJ/m^3^)
Specimen (0°)	186.48 ± 6.22	1.476 ± 0.447	206.33 ± 73.16
Specimen (±15°)	200.58 ± 6.93	0.879 ± 0.154	124.70 ± 25.92
Specimen (±30°)	174.83 ± 9.11	2.273 ± 0.111	335.42 ± 12.81
Specimen (±45°)	176.16 ± 11.37	2.699 ± 0.555	386.55 ± 100.41
Specimen (±60°)	130.74 ± 8.12	0.414 ± 0.096	32.66 ± 2.84
Specimen (±75°)	90.91 ± 4.19	0.382 ± 0.019	17.61 ± 0.98
Specimen (90°)	70.86 ± 13.65	0.289 ± 0.074	11.55 ± 2.13

**Table 5:** Failure mode results of bearing test specimens.

Specimen Type	Sample 1	Sample 2	Sample 3	Sample 4	Sample 5
Specimen (0°)	B	B	B	B	B
Specimen (±15°)	B	B	B	B	B
Specimen (±30°)	B	B	B	B	B
Specimen (±45°)	B	B	B	B + N	B
Specimen (±60°)	N	N	N	N	N
Specimen (±75°)	N	N	N	N	N
Specimen (90°)	N	N	N	N	N

B: Bearing failure mode; N: net-tension failure mode.

## Data Availability

The original contributions presented in the study are included in the article; further inquiries can be directed to the corresponding author.
